# Supplementary material for: Identification of photoperiod-induced specific miRNAs in the adrenal glands of Sunite sheep (Ovis aries)
Source: Front Vet Sci. 2022 Jul 22;9:888207. doi: 10.3389/fvets.2022.888207 (PMC9354845; doi:10.3389/fvets.2022.888207)
Supplement: Supplementary file 1 [file Data_Sheet_1.ZIP › Supplementary file/miRDeep2 file.zip!/miRDeep2 file/Know_Structure/LP42A2/oar-mir-26a.pdf]

miRBase precursor : oar-mir-26a
Total read count : 1119590
oar-miR-26a read count : 1119395
remaining reads : 195

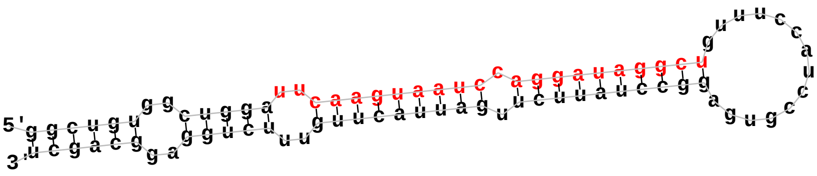

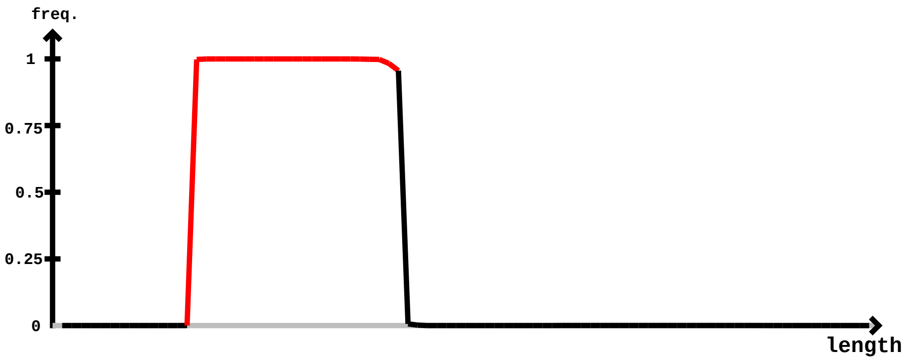

oar-miR-26a

```
5'- ggcuguggcuggauucaaguaauccaggauaggcuguuuccauccgugaggccuauucuugauuacuuguuucuggaggcagcu   -3'    exp
    ((((((..(((((..(((((((((.((((((((((..............)))))))))).)))))))))..)))))..))))))    reads      mm         sample
```

sequence	reads	mm	sample
............auucaaguaauccaggauagg...	1	0	seq
............auucaaguaauccaggauaggc..	5	0	seq
............Guucaaguaauccaggauaggcu...	23	1	seq
............auucaaguaauccaggauaggcu...	140	0	seq
.............uucaaguGauccaggau..	1	1	seq
.............uucaaguaauccaggaA..	22	1	seq
.............uucaUguaauccaggau..	1	1	seq
.............uucaaguaauccaggau..	539	0	seq
.............uucaaguaauccaggaG..	11	1	seq
.............uucaaAuaauccaggau..	2	1	seq
.............uAcaaguaauccaggau..	2	1	seq
.............uucaGguaauccaggau..	3	1	seq
.............uucaGguaauccaggaua...	4	1	seq
.............uucaaguaauccaggaua...	873	0	seq
.............uucaaguaauccaggauag..	732	0	seq
.............uucaaguaauccaggauaC..	3	1	seq
.............uucaaguaauccaggauagg...	16649	0	seq
.............uucaaguaauccaggauaggc..	30213	0	seq
.............uucaaguaauccaggauaggcu...	1060907	0	seq
.............uucaaguaauccaggauaggcug..	5094	0	seq
.............uucaaguaauccaggauaggcugu...	1904	0	seq
.............uucaaguaauccaggauaggcuguG..	5	1	seq
.............uucaaguaauccaggauaggcuguu..	24	0	seq
.............uucaaguaauccaggauaggcuguuu...	6	0	seq
..............ucaaguaauccaggaua...	1	0	seq
..............ucaaguaauccaggauag..	2	0	seq
..............ucaaguaauccaggauagg...	16	0	seq
..............ucaaguaauccaggauaggc..	64	0	seq
..............ucaaguaauccaggauaggcu...	1988	0	seq
..............ucaaguaauccaggauaggcug..	18	0	seq
..............ucaaguaauccaggauaggcugu...	8	0	seq
..............ucaaguaauccaggauaggcuguu..	1	0	seq
...............caaguaauccaggauagg...	1	0	seq
...............caaguaauccaggauaggc..	4	0	seq
...............caaguaauccaggauaggcu...	96	0	seq

oar-miR-26a

ggcuguggcugga**uucaaguaauccaggauaggcu**guuuccauccgugaggccuauucuugauuacuuguuucuggaggcagcu			
................aaguaauccaggauagg...	1	0	seq
................aaguaauccaggauaggc..	2	0	seq
................aaguaauccaggauaggcu...	24	0	seq
................aaguaauccaggauaggcug..	1	0	seq
.................aguaauccaggauaggcu...	2	0	seq
..................guaauccaggauaggcu...	2	0	seq
....ccgugaggccuauucuugauuacu.................	1	0	seq
....aggccuauucuugauuacuugu..............	1	0	seq
....aggccuauucuugauuacuuguu.............	1	0	seq
....ggccuauucuugauuacuuguu.............	1	0	seq
....ccuauucuugauuacuug...............	1	0	seq
....ccuauucuugauuacuuguu.............	10	0	seq
....ccuauucuugauuacuuguuu............	92	0	seq
....ccuauucuugauuacuuguuuc...........	85	0	seq
....ccuauucuugauuacuuguuucu..........	2	0	seq
....cuauucuugauuacuuguuuc...........	1	0	seq
